# Learning from EMG: semi-automated grading of facial nerve function

**DOI:** 10.1007/s10877-021-00793-y

**Published:** 2022-01-06

**Authors:** Magdalena Holze, Leonhard Rensch, Julian Prell, Christian Scheller, Sebastian Simmermacher, Maximilian Scheer, Christian Strauss, Stefan Rampp

**Affiliations:** 1 Department of Neurosurgery, University Hospital Halle (Saale), Halle, Germany; 2 Present Address: Department for General, Visceral and Transplantation Surgery, University Hospital Heidelberg, Heidelberg, Germany

**Keywords:** House–Brackmann, Facial EMG, Facial nerve function, Grading system, Interobserver variability, Vestibular schwannoma

## Abstract

The current grading of facial nerve function is based on subjective impression with the established assessment scale of House and Brackmann (HB). Especially for research a more objective method is needed to lower the interobserver variability to a minimum. We developed a semi-automated grading system based on (facial) surface EMG-data measuring the facial nerve function of 28 patients with vestibular schwannoma surgery. The sEMG was recorded preoperatively, postoperatively and after 3–12 months. In addition, the HB grade was determined. After manual selection and preprocessing, the data were subjected to machine learning classificators (Logistic regression, SVM and KNN). Lateralization indices were calculated and multivariant machine learning analysis was performed according to three scenarios [differentiation of normal (1) and slight (2) vs. impaired facial nerve function and classification of HB 1-3 (3)]. The calculated AUC for each scenario showed overall good differentiation capability with a median AUC of 0.72 for scenario 1, 0.91 for scenario 2 and multiclass AUC of 0.74 for scenario 3. This study approach using sEMG and machine learning shows feasibility regarding facial nerve grading in perioperative VS-surgery setting. sEMG may be a viable alternative to House Brackmann regarding objective evaluation of facial function especially for research purposes.

## Introduction

Vestibular schwannomas are brain tumors located in the cerebellopontine angle (CPA) which affect the facial nerve in several ways. Its location may lead to compression of the facial nerve with potential impact on its function [1]. Furthermore, VS surgery itself may damage the facial nerve and results in postoperative facial palsy. This may have consequences for patient health and quality of life [2–4].

Currently, the House-Brackmann facial nerve grading system (HBGS) is the standard method to assess facial nerve function. First introduced in 1983 it was endorsed as the main grading system by the Facial Nerve Disorders Committee of the American Academy of Otolaryngology-Head and Neck Surgery in 1984 [5]. However, since then its reliability and suitability has been discussed constantly [6, 7].

Next to HBGS, many other facial grading systems have been developed to classify facial nerve function. The Yanagihara grading system for instance was already developed and applied in 1976 as a regional classification system measuring ten separate aspects of facial function summarized into a total score [8]. New systems like the Nottingham [9], the Sydney [10], the Sunnybrook Facial Grading Systems [11] or the MoReSS [12] as a modification of the HBGS were evolved in the following years.

Although all these grading systems and modifications have been developed, the common issue of observer subjectivity remains a significant source of variability and inaccuracy [6, 13]. Smith et al. compared multiple leading systems, including HBGS, and found similar interobserver variation in all of them [14]. The HBGS was updated in 2009 by the Facial Nerve Disorders Committee to incorporate a regional scoring scale and to limit interobserver variability [15]. Nevertheless, this resulted in only moderate improvements and limited impact on clinical practice as Scheller et al. [6] showed in 2017. Despite its easy application and ability to identify clinically relevant facial palsy, the limited interrater reliability of also the updated HBGS impacts its sensitivity and robustness required to evaluate smaller differences.

These remaining issues can also be seen in ongoing publication of novel approaches utilizing more modern techniques like video-analysis, e.g. as developed by Banks et al. [16, 17]. Their system is based on subject video assessment, albeit using a detailed and robust approach, considering static, movement and synkinesis parameter. However, availability and simple operability of these systems remain problematic which prevents their wider adoption as a standard facial grading system for clinical routine.

The ability to document more subtle differences in facial nerve function with low interrater variability has limited relevance for clinical practice. However, this aspect becomes especially important for research. Large interrater variability significantly constrains development and optimization e.g. of neuromonitoring approaches and pharmacotherapy [18–20]. The surface electromyography (sEMG) based method in the current study is intended to meet such requirements of low examiner-dependence with limited added measuring effort.

Our approach evaluates facial nerve function using surface sEMG. sEMG measures the activation of muscle fibres which is related to the level of contraction of motor units. Ryu et al. and Kim et al. showed that there is indeed considerable correlation between sEMG and clinical assessment tools (House–Brackmann scale, Yanagihara grading system, Sunnybrook facial grading system) [21, 22]. Despite these studies on sEMG to assess facial function, there is a lack of studies on neurosurgical patients undergoing VS surgery.

Consequently, there is still a need for a new objective measurement technique for clinical research in this area with the aim of improving surgical treatment, specifically regarding facial nerve function. Our exploratory study shows that sEMG may be a potential solution.

## Method

### Patients

Twenty-eight patients undergoing elective surgery for vestibular schwannoma were recruited for the study from April 2017 until July 2018 in the order in which the patients had their surgery appointment or control investigation.

Inclusion criteria were (1) indication for vestibular schwannoma surgery due to suspected VS, independent of primary tumor or recurrence, tumor size and postoperative histological diagnosis and (2) adult age.

The exclusion criteria were (1) neurofibromatosis, (2) tumor at a different location with the consequence of other surgical procedures (e.g. ependymoma/ metastasis of the posterior cranial fossa with similar symptoms) and (3) preoperative facial palsy caused by central nerve disease.

The study was positively reviewed by the institutional review board of the University Hospital Halle (Saale). All patients gave their written informed consent to participate in the study.

### Recordings

sEMG was recorded with a Grass-Telefactor 15LT biosignal amplifier (West Warwick, Rhode Island) which is also used for standard procedures, such as continuous EMG and intraoperative monitoring (IOM) at the Department of Neurosurgery at the University Hospital Halle (Saale) and already described in earlier studies [18].

**Fig. 1:**
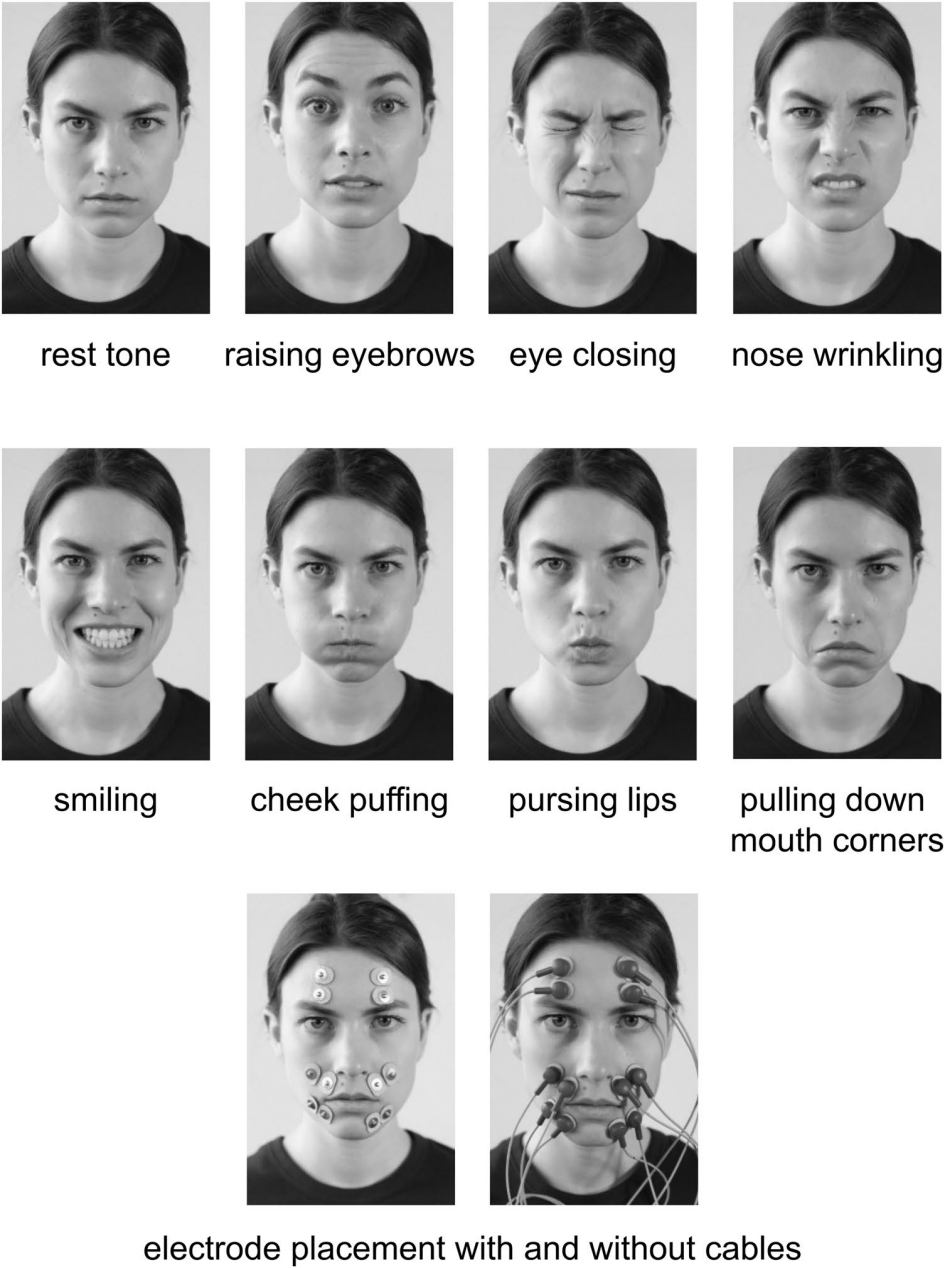
Demonstration of the seven different facial poses and electrode placement

sEMG was recorded at defined time points: 1 day before surgery, between the fifth and tenth postoperative day and at follow-up evaluations at 3, 6 or 12 months after surgery. EMG amplitudes from seven different facial poses were recorded (Fig. 1). These poses where chosen to ensure high comparability between measurements and clinical evaluation. They were the same movements as those used in the examination of the HBGS. In addition, they show the highest EMG activity of the representative facial nerve innervated muscles [23, 24].

One electrode pair was placed on the forehead, one on the nasolabial fold and one was positioned underneath the lips on the lateral chin (Fig. 1). A ground electrode was positioned on the right wrist. Before application of the electrodes, the skin was cleaned with alcohol swipes to reduce impedance.

Before the measurement, the movements were demonstrated by the examiner and the patients were encouraged to practice every movement to guarantee correct performance. The patients were also motivated to perform the movements as strongly as they could to record the maximum muscle activity. EMG activity was recorded during three repetitions for each pose to capture intraindividual variability. The tension time was about 1 s, the relaxation time 3 s. All measurements were done by the same person (author MH) who was fully familiar with the measurement method and instrument to prevent the effect of variability due to different examiners.

### Assessment scale

The House–Brackmann Grade was determined by a single examiner (author JP) with the aid of photographs of all poses. These were routinely taken at all defined time points.

### Data processing

**Fig. 2:**
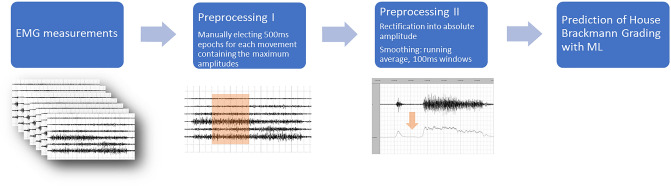
Overview of the methodological and analytical steps

As a first step, 500 ms epochs containing the maximum amplitudes of each movement were manually selected. (in-house software). Data were then rectified to obtain the absolute amplitude and smoothed with 100ms window running average. Calculation of the 95th percentile then yielded a single EMG amplitude value per channel. An overview of the various methodological and analytical steps in chronological order is shown in Fig. 2.

### Statistical analysis

From the ipsi- and contralateral EMG amplitudes of the orbicular oculi, nasalis and orbicular oris muscles and each repetition, lateralization indices (LI) were calculated according to the formula:

$${\text{LI}} = {{\left( {{\text{EMG}}\_{\text{ipsi}} - {\text{EMG}}\_{\text{contra}}} \right)} \mathord{\left/ {\vphantom {{\left( {{\text{EMG}}\_{\text{ipsi}} - {\text{EMG}}\_{\text{contra}}} \right)} {\left( {{\text{EMG}}\_{\text{ipsi + EMG}}\_{\text{contra}}} \right)}}} \right. \kern-\nulldelimiterspace} {\left( {{\text{EMG}}\_{\text{ipsi + EMG}}\_{\text{contra}}} \right)}}.$$

**Fig. 3:**
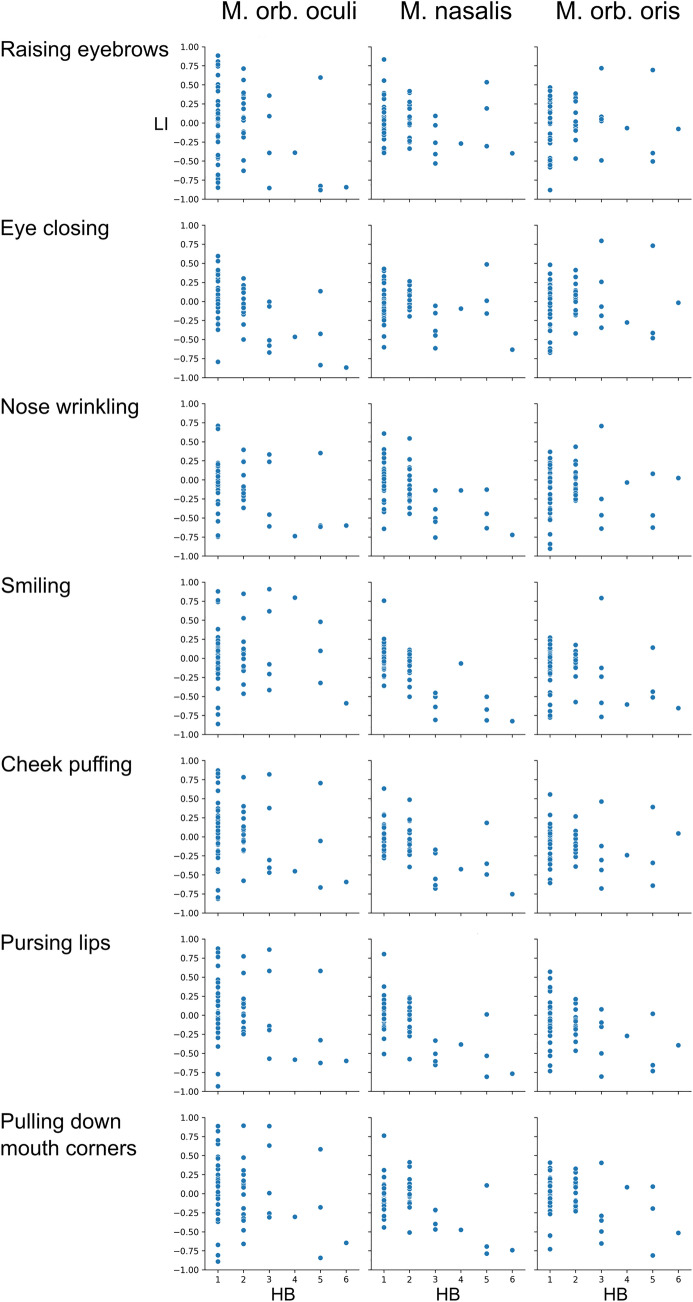
Overview of the lateralization indices (LI) of the individual muscle groups for all seven movements for the respective HB grades. A LI of one would result from sEMG-activity only ipsilateral to the operated side, respectively a LI of − 1 would show only contralateral sEMG

While absolute EMG amplitudes likely contain information about facial nerve function, intraindividual asymmetries of ipsi- vs. contralateral facial muscles strongly influence clinical evaluation of HB grades. Furthermore, LI take interindividual variability of facial muscle movement as well as small differences in recording setup into account. Due to the limited sample size we only used LI for analysis and did not additionally include absolute values. Furthermore, most HB grades were in the range of HB 1–3, which are characterized by subtler changes which might be more apparent by comparing ipsi- to contralateral EMG intraindividually (overview shown in Fig. 3).

Multivariate machine learning analysis was performed with scikit-learn version 0.24.2 (https://scikit-learn.org/), applying three algorithms: Logistic Regression, support vector machines (SVM using gamma = 1/number of features) and k nearest neighbor classification (KNN using k = 5 and distance weighting of neighbors). The aim was to take advantage of an automatic learning system which is able to recognize patterns between input and output data and to make predictions based on this process. To evaluate the viability of estimating HB grades from EMG data, we evaluated three classification scenarios: (1) Differentiation of normal vs. impaired facial nerve function (HB 1 vs. HB 2–6), (2) normal or slight vs. moderate impairment (HB 1–2 vs. HB 3–6) and (3) classification of HB grades 1–3. Further differentiation of individual classification of each possible HB grade (1–6) were not evaluated due to the limited sample size. Consequently, scenarios 1 and 2 represented a binary classification problem, whereas scenario 3 aims at multi-class classification. For the latter, the ordinal scaling of HB grades was not taken into account; multinomial classification was attempted. Due to the low sample size we did not optimize parameters of the three algorithms.

Cross-validation for training and testing utilized a “leave one out” strategy. This means that during each training-testing run, data from all patients except one were used for training, and testing was performed using the remaining. This was repeated until data from each patient had been left out and used for testing once, which aims to limit overfitting.

This training aims to let the respective algorithm learn the pattern between in- and output data, which can then be applied to estimate output data associated with never seen input data. The degree of correct estimated output data is then used to evaluate the performance. During the testing, the corresponding HB grades were estimated based on the learned patterns and compared to the actual HB grades.

Classification results, respectively class probabilities of only this testing split were accumulated and compared to the true clinical HB grades for estimation of performance. We calculated receiver-operator characteristic (ROC) and area-under-the-curve (AUC) values for scenarios 1 and 2. For scenario 3, the multiclass “one versus one” AUC was calculated, which computes the average AUC of all possible pairwise combinations of classes. Due to the largely imbalanced dataset with most patients with lower HB grades, we preferred AUC over accuracy due to the lower albeit still present susceptibility to this issue.

Additionally, an analysis was carried out to determine the over- and underestimation of each classifier compared to the clinical examined HB (for this purpose, only the test data from HB 1–3 of the leave-one-out strategy was used).

## Results

### Baseline data

Overall, 28 patients were recruited for sEMG-measurement. Mean age was 50 years (range 23–77); 75% were female. Mean tumor size was Koos 3 (range 1–6) [25] and 75% of the tumors were located on the left side. The postoperative histological examination revealed vestibular schwannoma in 24 patients (86%) and other histologies in four cases (14%, three meningiomas and one intermedius neurinoma). In a total of 59 measurements, 30 times a clinical HB 1, 17 times a clinical HB 2, 5 times a HB 3, 3 times a HB 4 and 2 times a HB 5 was evaluated. There were no HB grade 6 measurements. In two measurements (1 preoperative and 1 postoperative, each from different patients) the corresponding clinically evaluated HB was missing.

### Participant flow

There were no patient dropouts before preoperative data acquisition. In case of three patients, preoperative sEMG, in two patients the first postoperative sEMG and in 18 patients the long-term measurements were missing because of logistical reasons and limitation of the follow-up period. A total of 23 patients underwent both pre- and postoperative measurements, eight of them additionally during follow-up. Regarding House–Brackmann assessment, one preoperative evaluation was missing and one of the follow-ups.

In two cases of preoperative measurements, data of single repetitions and complete movements were missing because the patient could not perform the movement correctly or strong enough to achieve a representative EMG amplitude for further analysis. In one case smiling and blowing out cheeks was not possible, wrinkling the nose was accomplished just a single time. In the other case smiling was accomplished just one time instead of the three required trials. Such missing values were replaced with either the values of the available single measurement (nose wrinkling), so that there was virtually no mean value or the average of all other movements (in case of completely missing data of a movement). This procedure guarantees that the missing values are replaced by similar numbers instead of e.g. leaving those values out or by zeroing the missing values, which would distort the learning procedure.

### Estimation of facial nerve function

The first and second scenarios showed the following AUC depending on each classificator: Evaluating the differentiation of normal vs. impaired facial nerve function (HB 1 vs. HB 2–6) resulted in an AUC of 0.76 (Logistic Regression), 0.68 (SVM) and 0.73 (KNN). Differentiation of normal or slight vs. moderate impaired facial nerve function (HB 1 and 2 vs. HB 3–6) showed an AUC of 0.88 (Logistic Regression), 0.97 (SVM) and 0.89 (KNN). The multiclass AUC of HB 1, 2, and 3 carried out for scenario 3 yielded in an AUC of 0.74 (Logistic Regression), 0.70 (SVM) and 0.78 (KNN) each.

In addition, under- and overestimation by each algorithm were analyzed with the test data of HB 1–3, scenario 3. Logistic Regression was concordant in 66% and underestimated in 20% of the cases, i.e. it predicted a “better” HB grade than the actual clinical HB. There were 14% overestimated cases by Logistic Regression. SVM showed a concordance of 63% and predicted a “better” HB than it was clinically evaluated in 36% of cases, and it predicted a “worse” HB in 2%. For KNN the concordance was 66%, underestimation 27% and the overestimation 7%.

## Discussion

To the best of our knowledge this is the first study to use sEMG and machine learning as an objective assessment tool before and after VS-surgery for comparison with HBGS.

Our results show that there is an overall good differentiation capability of the estimated HB with machine learning compared to the clinical determined HB and clinically relevant differences can be distinguished.

### Machine learning vs. clinical assessment

HB grades estimated by machine learning algorithms showed a good differentiability and especially clinically relevant gradings of facial nerve function could be distinguished. Differentiation between slight (HB 1–2) and moderate impaired facial nerve function (HB 3–6, scenario 2) showed with 0.88–0.97 the best results. In addition, the AUC of scenario one (0.68–0,73, differentiation of normal vs. impaired facial function) confirms an overall acceptable performance and motivates to further studies with larger data sets beyond the exploratory character of our current investigation.

A general concern when comparing the estimated results with the clinical assessment is the lack of a true gold standard. A main question of our new approach is whether it categorizes the facial function better and more precisely than the previous grading systems. While HB is the de-facto clinical standard, its evaluation is subjective and suffers from considerable interrater variability [6]. We tried to minimize interobserver variability as much as possible by having the same investigator determine the HB grade. A high or even perfect correlation between our method and HB is not expected nor desirable as this would require reproducing the subjective variability.

Logistic regression, SVM and KNN showed different degrees of under- and overestimation i.e. deviation from the clinically rated HB. For example, an examined HB 1 was partially rated as HB 2 (overestimation for Logistic regression of 14%, for SVM of 2% and for KNN of 7%) and vice versa, a HB 2 as HB 1 (Logistic regression underestimated in 20%, SVM in 36% and KNN in 27% of the cases). An underestimated HB could be caused by high EMG activity, despite already present palsy, caused by synkinesis and attempted compensation by the opposite side. An overestimated HB on the other hand could be due to subtle asymmetries in muscle activity which is only detectable by EMG but not yet visible for the investigator. Alternatively, of course, fatigue or limited patient compliance are further explanations for overestimated HB.

In order to additionally illustrate the advantages of the machine learning approach regarding interrater reliability, we evaluated concordance rates with the clinical examiner. Logistic regression and KNN showed concordance with the clinical examiner in 66% respectively, SVM in 63%. After training, the classification results provided by all three approaches are guaranteed to be reproducible with the same input data, leaving factors of the sEMG recording, e.g. patient compliance, data quality, etc. as the only sources of variability.

In comparison, the study by Scheller et al. showed an overall concordance of only 36% and interobserver variability by one degree of 45%, by two degrees of 17% and by three degrees of 1% [6]. Reproducibility of clinical evaluation was not investigated. The study used patient photos for evaluation, i.e. the reported concordance rates already exclude variability from the clinical investigation itself. Overall, although our concordance data is restricted to the lower HB grades, our results suggest that a similar or improved performance with high reproducibility using sEMG should be viable.

### Alternative methods

Many facial nerve grading systems have been developed in the past [5, 8–12], most of them with the goal to provide clinical assessment tools. All these systems share the approach that the clinical observer is the central evaluating component. The problem of subjectivity and interobserver variability is therefore inherent to such assessment tools and can only be improved to a certain extent [6, 7, 13, 14]. Their strength consists in the easy handling and practicability in clinical routine.

Recently, the interest in computer-based systems and sEMG as clinical tools has grown [21, 22]. However, such systems are not yet available everywhere due to the required technical equipment and the rather limited ease of practical implementation and application [11]. Therefore, clinical routine, but also randomized controlled trials have to rely on such subjective assessment tools. In 2017, Scheller et al. stressed this issue as a major problem regarding their randomized multi-center phase III trial on the efficacy of prophylactic nimodipine treatment in vestibular schwannoma (VS) surgery [19]. Potentially, they argued, the large interrater variability of their HB grades may have obfuscated smaller effects of vasoactive treatment on postoperative facial nerve function and hearing.

### sEMG for grading of facial nerve function

In patients with vestibular schwannoma surgery, other studies on the use of sEMG for grading of facial function are not available. The general approach of sEMG-based evaluation of facial function outside the surgical field however has gained interest. For example Ryu et al. [21] investigated sEMG as an assessment tool for facial palsy. They conducted sEMGs on 50 patients with peripheral facial nerve palsy at different points in time (1, 3–4 and 5–6 weeks after onset) and analyzed the correspondence of the individual facial regions with nerve conduction studies (NCS) and several clinical assessment systems including HB. Placement and number of electrodes in their study were similar to ours and the facial movements were almost identical. They report a good correspondence to NCS and clinical assessment. A study by Kim et al. [9] showed similar results when comparing sEMG recordings of 21 patients with peripheral nerve palsy with three common clinical assessment scores (HBGS, Sunnybrook Facial Grading System and Yanagihara grading system).

Our results are in line with these studies and further support sEMG as a useful tool for the assessment of facial nerve function also in a surgical setting. Above and beyond, we developed an alternative grading method, with which an objective classification into different degrees of severity is possible based on the sEMG.

Another advantage of sEMG is the objective comparability of both face halves of individual patients. This might be useful in case of synkinesis. Bernardes et al. [26] showed that there is significant synkinesis even in healthy patients between both halves. This suggests a need for an objective but still comparable parameter which can evaluate the healthy and affected half of the face separately. In comparison, by evaluating the HBGS, the face of the patient is only visible to the examiner as a whole. Subjective visual assessment of the degree of synkinesis and taking this into account for facial function grading may be challenging and may be less reliable. Our current implementation potentially suffers from the effect of synkinesis. However, as separate measurements are available, it is conceivable to extend the procedure to take synchronization between face halves into account and thus limit the impact of synkinesis or separately take this into account for grading.

Regarding the optimal electrode number and placement, Schumann et al. [23] conducted research on facial muscle activation patterns in healthy individuals with sEMG. They showed objective and statistically representative sEMG patterns of symmetric facial muscle function in 30 healthy subjects. Their primary objective was to contribute reference data for neurological examination guidelines with the focus on the optimal number and positioning of the electrodes. Recordings used 48 electrode pairs compared to six electrodes in our study. An advantage of a high number of electrodes might be the more precise detectability of the distribution of muscle activity. However, as the authors mention themselves, the high number of electrodes complicates evaluation and interpretation in clinical routine. Fewer electrodes with however optimal placement may be sufficient for evaluation due to practical, simple, and thus reproducible positioning. To this end, further studies with the aim of finding optimal electrode placement are required.

### Limitations

A major limitation of our study is the comparably small sample. This likely led to a limited accuracy of performance estimation and facilitated overfitting. To minimize any dependence of the training and test data the leave-one-out approach was chosen. This strategy counteracts as well the possible confounder of the time course as the course of disease, if the data occur from the same patient which cannot appear in the training and test split in one run. The performance of all classifiers would clearly benefit from a much larger training sample. With a larger and more representative dataset, acquired in future studies, an improved analysis could also use the absolute EMG amplitudes instead of or additionally to the lateralization indices. In summary, the reported results probably overestimate the performance in the current data. However, they provide a proof-of-principle to conduct larger studies with potentially overall better performance of the method.

A limitation in the same vein is that not all HB-grades were equally represented. This however reflects the clinical presentation of facial function before and after surgery [27]. Because of this issue we have evaluated the three scenarios and also limited scenario 3 to only HB 1, 2 and 3 to counteract this imbalance. Nevertheless, we believe the reported AUC values demonstrate the viability of our approach but are likely optimistic. Ideally, a larger dataset should be balanced in terms of HB grades for optimal training success.

Although equipment to record sEMG should be available in many neurosurgical centers, the measuring effort itself including placing the electrodes is another disadvantage. In addition, the patient needs to sit in an upright position and must be able to collaborate during the time-consuming measurement. Therefore, the procedure may in fact mostly be viable for research purposes—corresponding to our study aims.

### Conclusions

In summary, this pilot study shows that sEMG can be used in principle to grade facial nerve function and may be a potential alternative or addition to HBGS, primarily for scientific investigations. Our results showed overall good differentiation capacity between clinically relevant HB-ranges. Furthermore, our machine learning based approach is an automated method and yields reproducible results.

Given the need for even more accurate and precise classification systems our findings make an important contribution towards more objectivity. Because of the setting as a pilot study and limitations regarding sample size and capturing all HB ranges further studies are planned. Above all, more data in general as well as the different degrees of severity of facial palsy are to be represented equally, in order to improve and accurately evaluate performance.

## References

[1] Cushing H (1917). Tumors of the nervus acusticus and the syndrome of the cerebellopontine angle.

[2] Ho AL, Scott AM, Klassen AF, Cano SJ, Pusic AL, Van Laeken N (2012). Measuring quality of life and patient satisfaction in facial paralysis patients: a systematic review of patient-reported outcome measures. Plast Reconstr Surg.

[3] Coulson SE, O’Dwyer NJ, Adams RD, Croxson GR (2004). Expression of emotion and quality of life after facial nerve paralysis. Otol Neurotol.

[4] Bateman N, Nikolopoulos TP, Robinson K, O’Donoghue GM (2000). Impairments, disabilities, and handicaps after acoustic neuroma surgery. Clin Otolaryngol Allied Sci.

[5] House JW, Brackmann DE (1985). Facial nerve grading system. Otolaryngol Head Neck Surg.

[6] Scheller C, Wienke A, Tatagiba M, Gharabaghi A, Ramina KF, Scheller K (2017). Interobserver variability of the House-Brackmann facial nerve grading system for the analysis of a randomized multi-center phase III trial. Acta Neurochir (Wien).

[7] Kang TS, Vrabec JT, Giddings N, Terris DJ (2002). Facial nerve grading systems (1985-2002): beyond the House-Brackmann scale. Otol Neurotol.

[8] Yanagihara N. Grading of facial palsy. In: Fisch U Facial nerve surgery. Proceedings of the Third International Symposium on Facial Nerve Surgery; Zurich; 1976. pp. 533–5 Birmingham; Aesculapius Publishing Company 1977.

[9] Murty GE, Diver JP, Kelly PJ, O’Donoghue GM, Bradley PJ (1994). The Nottingham system: objective assessment of facial nerve function in the clinic. Otolaryngol Head Neck Surg.

[10] Coulson SE, Croxson GR, Adams RD, O’Dwyer NJ (2005). Reliability of the “Sydney,“ “Sunnybrook,“ and “House Brackmann” facial grading systems to assess voluntary movement and synkinesis after facial nerve paralysis. Otolaryngol Head Neck Surg.

[11] Ross BG, Fradet G, Nedzelski JM (1996). Development of a sensitive clinical facial grading system. Otolaryngol Head Neck Surg.

[12] de Ru JA, Braunius WW, van Benthem PP, Busschers WB, Hordijk GJ (2006). Grading facial nerve function: why a new grading system, the MoReSS, should be proposed. Otol Neurotol.

[13] Fattah AY, Gurusinghe AD, Gavilan J, Hadlock TA, Marcus JR, Marres H (2015). Facial nerve grading instruments: systematic review of the literature and suggestion for uniformity. Plast Reconstr Surg.

[14] Smith IM, Murray JA, Cull RE, Slattery J (1992). A comparison of facial grading systems. Clin Otolaryngol Allied Sci.

[15] Vrabec JT, Backous DD, Djalilian HR, Gidley PW, Leonetti JP, Marzo SJ (2009). Facial nerve grading system 20. Otolaryngol Head Neck Surg.

[16] Banks CA, Bhama PK, Park J, Hadlock CR, Hadlock TA (2015). Clinician-graded electronic facial paralysis assessment: the eFACE. Plast Reconstr Surg.

[17] Banks CA, Jowett N, Hadlock TA (2017). Test–retest reliability and agreement between in-person and video assessment of facial mimetic function using the eFACE facial grading system. JAMA Facial Plast Surg.

[18] Prell J, Rachinger J, Scheller C, Alfieri A, Strauss C, Rampp S (2010). A real-time monitoring system for the facial nerve. Neurosurgery.

[19] Scheller C, Wienke A, Tatagiba M, Gharabaghi A, Ramina KF, Ganslandt O (2016). Prophylactic nimodipine treatment for cochlear and facial nerve preservation after vestibular schwannoma surgery: a randomized multicenter Phase III trial. J Neurosurg.

[20] Scheller C, Wienke A, Wurm F, Simmermacher S, Rampp S, Prell J (2014). Neuroprotective efficacy of prophylactic enteral and parenteral nimodipine treatment in vestibular schwannoma surgery: a comparative study. J Neurol Surg A Cent Eur Neurosurg.

[21] Ryu HM, Lee SJ, Park EJ, Kim SG, Kim KH, Choi YM (2018). Study on the validity of surface electromyography as assessment tools for facial nerve palsy. J Pharmacopuncture.

[22] Kim JU, Lee HG, Jung DJ, Choi YM, Song BY, Yook TH (2013). A study on the correlation between surface electromyography and assessment scale for facial palsy. The Acupuncture.

[23] Schumann NP, Bongers K, Guntinas-Lichius O, Scholle HC (2010). Facial muscle activation patterns in healthy male humans: a multi-channel surface EMG study. J Neurosci Methods.

[24] Choi YM, Kim JU, Kim LH, Yook TH (2017). A study of the electrical properties of the buccal area using facial surface electromyography. The Acupuncture.

[25] Koos WT, Day JD, Matula C, Levy DI (1998). Neurotopographic considerations in the microsurgical treatment of small acoustic neurinomas. J Neurosurg.

[26] Bernardes DFF, Bento RF, Goffi Gomez MVS (2018). The contribution of surface electromyographic assessment for defining the stage of peripheral facial paralysis: flaccid or sequelae stage. Int Arch Otorhinolaryngol.

[27] Falcioni M, Fois P, Taibah A, Sanna M (2011). Facial nerve function after vestibular schwannoma surgery. J Neurosurg.

